# *Fusobacterium* Species in Osteoarticular Infections in Childhood—A Systematic Review with Data Synthesis and a Case Series in the Acetabular and Hip Joint Regions

**DOI:** 10.3390/idr17020030

**Published:** 2025-04-10

**Authors:** Heide Delbrück, Silvia Schröder, Tom Stapper, Sabine Schacht, Angeliki Pappa, Frank Hildebrand, Miriam Katharina Hertwig

**Affiliations:** 1 Department of Orthopaedics, Trauma and Reconstructive Surgery, University Hospital RWTH Aachen, Pauwelsstrasse 30, 52074 Aachen, Germany; 2 Center for Paediatric & Adolescent Medicine, Mönchengladbach Municipal Clinics, Elisabeth-Krankenhaus Rheydt, Hubertusstrasse 100, 41239 Mönchengladbach, Germany; 3 Department of Diagnostic and Interventional Radiology, University Hospital RWTH Aachen, Pauwelsstrasse 30, 52074 Aachen, Germany; 4 Department for Paediatrics and Adolescent Medicine, University Hospital RWTH Aachen, Pauwelsstrasse 30, 52074 Aachen, Germany

**Keywords:** *Fusobacterium*, children, paediatric, osteoarticular infections, musculoskeletal infections, bone and joint infections, osteomyelitis, septic arthritis

## Abstract

**Background**: In paediatric osteoarticular infections, microorganism detection is unsuccessful in up to 55% of cases, which is not satisfactory for targeted antibiotic therapy. In particular, anaerobic fusobacteria may be underdiagnosed owing to a lack of knowledge about their properties. **Methods**: Based on three of our own cases and a systematic literature review regarding paediatric osteoarticular fusobacterial infections, we extracted characteristic variables and synthesised them in terms of frequencies and mean comparisons. We followed the CARE and PRISMA guidelines. **Results**: In our three patients with hip area infections (aged 11, 12, and 16 years; two males and one female; two with *Fusobacterium nucleatum* [FNU] and one with *Fusobacterium necrophorum* [FNE]), we only detected FNU with PCR. The patient with an FNE infection showed a septic and protracted course with six surgical interventions and secondary coxarthrosis during the follow-up. The FNU infections were milder and healed without sequelae. In the literature, there are no articles with more than 3 cases; overall, we identified 38 case reports and 3 case series with a total of 45 patients. Across all synthesised cases (73% boys), the age was 9.2 ± 4.1 years. Most patients (42%) were affected by hip joint arthritis, with or without accompanying acetabular or femoral osteomyelitis, followed by knee joint arthritis, with or without osteomyelitis, in 24% of patients. In 49% of cases, there was an ear, nose, and throat focus. Depending on the affected structure, arthrotomy (33%), arthroscopy (11%), bone (24%), and soft tissue (9%) debridement were performed, with 34% of the procedures having to be performed several times. Penicillins, metronidazole, and clindamycin were the most used antibiotics. In 32 cases (71%), the authors reported healing without sequelae. **Conclusions**: When samples are collected in the operating theatre for paediatric osteoarticular infections, orthopaedic surgeons should also ensure correct anaerobic sampling and consider the possibility of performing PCR. A typical child with an osteoarticular fusobacterial infection is a boy of approximately 10 years of age with an infection in the hip area and a previous infection in the ENT area.

## 1. Introduction

In the prospective multicentre European Union Childhood Life-threatening Infectious Diseases Study (EUCLIDS) with 380 patients, a causative organism in paediatric osteoarticular infections was only detected in 65% (247/380) of the patients. *Staphylococcus aureus* was identified in 57.1% (141/247) of the microbiologically confirmed cases, followed by Group A *Streptococcus* (18.2%) and *Kingella kingae* (8.9%) [1]. In literature reviews, the rate of unsuccessful microorganism detection is as high as 55% [2]. However, this aspect is of major importance for targeted antibiotic therapy in light of the possible long-term consequences of osteoarticular infections in children, such as growth disorders, bone deformities, joint destruction, restricted range of motion (ROM), osteoarthritis, and/or intermittent exacerbation of chronic osteomyelitis.

*Fusobacterium* species are anaerobic, Gram-negative bacteria that occur physiologically in the oral cavity, gastrointestinal tract, and female genital tract. *Fusobacterium necrophorum* (FNE) and *Fusobacterium nucleatum* (FNU) are among the most common species. The severity of these infections is highly variable, ranging from uncomplicated to Lemierre’s syndrome. [3,4] Lemierre’s syndrome is a serious disease caused by FNE. The infection takes place in the throat and spreads through septic thrombosis of the tonsillar and internal jugular veins. It is complicated by septic emboli in several places, such as the lungs, joints, and bones [5]. A rare abdominal variant of Lemierre’s syndrome with liver abscesses associated with suppurative portal vein thrombosis (pylephlebitis) caused by FNE is also described [6]. However, according to a systematic review by Jaber et al. [7], the occurrence of the gastrointestinal variant of Lemierre’s syndrome has not yet been described in children.

*Fusobacteria* are rarely mentioned in current textbooks as a cause of paediatric osteoarticular infections [8]. However, these infections may be more common than previously assumed because some *Fusobacteria* are difficult to culture and, therefore, are possibly underdiagnosed [9]. Polymerase chain reaction (PCR)-based diagnostics might help to reduce the rate of unsuccessful detection of these microorganisms [10].

Although these are infections that require interdisciplinary treatment, the orthopaedic surgeon plays a key role in obtaining and sending tissue for microbiological examinations as well as local infection therapy. Here, we present a case series with three patients treated in our department and a systematic literature review that is intended to describe the characteristics of paediatric musculoskeletal fusobacterial infections. We aim to increase the awareness of these causative microorganisms; an endeavour that could help to further close the gap of unidentified germs.

## 2. Methods

### 2.1. Case Series

The paediatric patients treated at our university hospital in 2023 and 2024 with orthopaedic consequences of fusobacterial infection were retrospectively described as case reports according to the Consensus-based Clinical Case Reporting (CARE) guidelines [11].

### 2.2. Methods of Systematic Literature Review

The systematic review was performed in accordance with the Preferred Reporting Items for Systematic Reviews and Meta-Analyses (PRISMA) guidelines [12].

#### 2.2.1. Search Strategy

MEDLINE (PubMed), Embase, Scopus, Epistemonikos, and CENTRAL were used as databases. The searches were performed on 8 August 2024. Alerts were used to regularly update the search for suitable publications until the data synthesis was completed on 24 November 2024. The study objective was transferred to the PICO (population, intervention, comparison, outcomes) scheme (Table 1).

The search terms are listed in Table 2. The detailed search strategy for each individual database can be found in Supplementary Table S1. The references of the identified articles were also searched for additional articles.

#### 2.2.2. Article Selection

Three reviewers (HD, MKH, and SiS) independently selected the articles to include in the systematic review. Duplicate elimination and title–abstract screening were performed with Rayyan software [13]. The articles selected for full-text screening were imported, sorted, and managed in Citavi (version 6.19.2.1, Swiss Academic Software GmbH, Wädenswil, Switzerland). Conflicts were resolved by consensus. The inclusion criteria used during article selection were broadly defined according to the review objectives (Table 3). No case reports with objective data were excluded in order to provide a broad overview of the existing literature and the described cases in this field.

#### 2.2.3. Data Extraction and Synthesis

The following parameters were extracted as indicated from the articles: age, sex, musculoskeletal region, presumed original focus, sepsis [14], fever at initial presentation (≥38 °C), additional lesions, C-reactive protein (CRP), the erythrocyte sedimentation rate (ESR), leucocyte count, duration of symptoms prior to initial presentation, *Fusobacterium* species, day of microorganism identification after sample collection, the blood culture results, microorganism identification in local tissue/fluid as well as by PCR, the type of orthopaedic diagnostic and therapeutic intervention, the main antibiotics and duration of their application after the microbe became known, the length of hospitalisation, the final outcome regarding orthopaedic lesions, and follow-up. Because the literature only contains case descriptions, these objective data were extracted for all described individual cases. The data were narratively synthesised with descriptive statistics and mean comparisons [15].

#### 2.2.4. Statistics

The statistical calculations were carried out with SPSS Statistics 29.0.0.0 (IBM Corp., Armonk, NY, USA). The *t*-test was used for metric data. The Shapiro-Wilk test and Levene’s test were used to determine whether the data had a normal distribution and homogeneity of variance. For non-normally distributed data, the Mann-Whitney U test was used for comparisons. To evaluate differences in frequencies, the chi-square test and Fisher’s exact test (if n = 5 in >20%) were used. For Fisher’s exact test, the Easy Fisher Exact Test Calculator was used (https://www.socscistatistics.com/tests/fisher/default2.aspx, accessed on 30 November 2024). A significant difference was defined as *p* < 0.05.

## 3. Results

### 3.1. Presentation of the Cases

Supplementary Table S2 presents a summary of our three cases.

#### 3.1.1. Patient 1

When this 10.5-year-old girl (36.4 kg, 1.44 m) was admitted to another hospital in mid-March 2023, she complained of right leg pain that had been unrelated to exertion for 10 days. Two weeks previously, she had experienced a single bout of severe diarrhoea without a fever > 38 °C. There were no other illnesses in her short- or long-term medical history.

**Figure 1:**
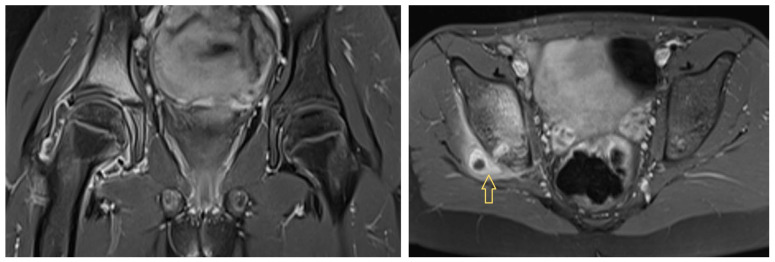
Initial magnetic resonance image of Patient 1 showing coxitis with a fluid path into the gluteal soft tissue. Arrow shows to the fluid path.

She had a limping gait and slightly restricted mobility of her right hip due to pain. Regarding inflammation, she had a CRP level of 45 mg/L and an ESR of 72 mm/h. An ultrasound examination revealed right hip joint effusion, and magnetic resonance imaging (MRI) performed immediately revealed signs of purulent coxitis and a fluid path from the dorsal acetabulum into the gluteal soft tissue, with abscess formation (Figure 1).

After transfer to our hospital, hip joint arthrotomy and irrigation with drain insertion were performed immediately. Her pain had improved significantly on the day after surgery (hospitalisation day [HD] 2), but it worsened again on the second postoperative day. A second surgical procedure was performed, including extensive capsular fenestration with irrigation and removal of the abscess on the dorsal acetabulum with the insertion of a gentamycin sponge.

Fourteen days after the start of treatment in our clinic, the patient was able to be transferred back to the hospital near her home. Her CRP level steadily decreased to normal levels, her ESR was 50 mm/h, and her pain slowly decreased. The daily ROM tests also revealed steady improvements, and her ROM finally reached a normal level.

The microbiological findings revealed no evidence of microbes until the day of transfer. Therefore, an additional eubacterial PCR was carried out on three samples, one of which presented a positive sequencing result for FNU: a 333-base pair (bp) sequence showed a 100% match with GenBank sequence CP071099. This finding was revealed 3 weeks after the first surgical procedure. The broad-range 16S rRNA gene PCR was carried out at the Institute for Microbiology and Hygiene in Regensburg, Germany [16].

During her stay in our clinic, the patient initially received 5 days of cefuroxime intravenously (1.2 g IV every 8 h), which was then escalated to vancomycin/meropenem (500 mg/750 mg IV every 8 h) due to a brief fever exacerbation. She then received ceftriaxone (1.2 g IV every 8 h) and clarithromycin (250 mg orally every 12 h) because *Mycoplasma pneumoniae* IgM was slightly elevated. After the diagnosis of a fusobacterial infection was confirmed, the patient received clindamycin (300 mg IV every 8 h) for 9 days, which was then switched to clindamycin 300 mg orally every 8 h for an additional 2 weeks.

During regular follow-up examinations in our outpatient clinic over approximately 18 months, a normal gait and normal ROM were observed in a symptom-free patient. The last MRI examination, 16 months after disease onset, revealed residual post-inflammatory structural irregularities in the dorsocranial right acetabulum without florid inflammatory changes in the pelvis and hip joints. There were no signs of femoral head necrosis.

#### 3.1.2. Patient 2

This 12.2-year-old boy (63 kg, 1.62 m) was transferred from another hospital to the paediatric oncology department of our university hospital at the beginning of May 2024. After intensive wakeboarding approximately 2 months previously, gluteal pain occurred on the right side, which ultimately did not improve even with ibuprofen. Furthermore, he had abdominal pain from time to time for 2 months, with intermittent fever up to 39 °C and loss of appetite for 2 weeks. No antibiotic prescription was documented by the GP during these abdominal pain episodes with fever.

The clinical examination did not reveal any movement restrictions in the hip joint at the time. The leucocyte count was normal, the CRP level was elevated at 40 mg/L, and the ESR was 114 mm/h. Nasopharyngeal swabs and blood cultures revealed unremarkable findings.

**Figure 2:**
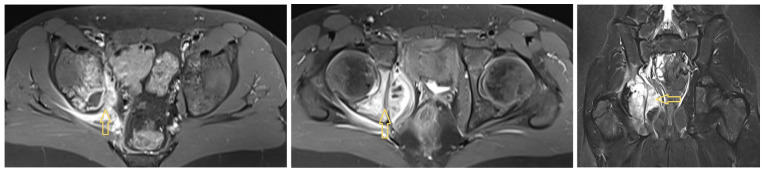
An osteodestructive process in the right os ilium/ischiadicum with cortical destruction. Arrow shows to the fluid path.

The external MR image revealed an extensive osteodestructive process in the right os ilium/ischiadicum with cortical destruction at the level of the medial acetabular wall and posterior acetabular pillar. There was melting at the level of the epiphyseal groove and subperiosteally on the medial acetabular wall, measuring up to 1.5 and 4.2 cm, respectively. The findings were primarily assessed as osteomyelitis; however, sarcoma was also discussed as a differential diagnosis (Figure 2).

The IV therapy with clindamycin (800 mg every 8 h) and ampicillin (1.5 g every 8 h) that had already been started in the transferring hospital was initially continued.

On the day after transfer (HD 2), the patient underwent the following procedure: fenestration of the abscess at the Y-joint on the right side using a 7 mm hollow reamer via the first window of the ilioinguinal approach, introduction of gentamycin-loaded calcium sulphate beads (STIMULAN Rapid Cure, Biocomposites Ltd., Staffordshire, UK) into the abscess cavity and the reaming channel, and refixation of the iliac crest apophysis and sartorius muscle via two 2.3 mm ICONIX™ All Suture Anchors (Stryker Corporation, Kalamazoo, MI, USA). Intraoperatively, pus was drained immediately after the hollow reamer was placed.

The bacteriological culture showed no evidence of bacteria or fungi after 2 weeks of incubation. Ten days after sample collection, we received the result of the eubacterial broad-range 16S rRNA gene PCR. The 333 bp sequence showed a 100% match with the following GenBank sequences: CP0841118 (FNU), CP077177 (*Fusobacterium animalis*), and CP056002 (*Fusobacterium vincentii*). The histopathological findings revealed a pronounced chronic florid inflammatory reaction with no evidence of any other disease.

Thirteen days after admission to our clinic, the patient was discharged with a normal CRP level and leucocyte count. His ROM for the right hip joint was still unrestricted. There was no more pain. Antibiotic therapy with oral clindamycin (600 mg every 8 h) was continued for 4 weeks after discharge from our hospital.

One month after the operation at our clinic, the control MRI revealed a clear regression of the inflammatory changes in the right ischium/acetabulum, with residual bone oedema. Furthermore, there was almost complete regression of periacetabular abscess formation and an irritation-free drill channel in the right pelvic blade. The clinical examination 6 months later revealed no abnormalities, and the patient was symptom-free.

#### 3.1.3. Patient 3

In mid-August 2024, a 16-year-old boy (84.5 kg, 1.75 m) with sepsis was transferred to our paediatric intensive care unit via helicopter with hypotension requiring catecholamines. He had a fever of up to 41 °C and a sore throat for 1 week. When the initial symptoms appeared, the patient was admitted to an external clinic, where he was discharged home with a suspected diagnosis of Epstein–Barr virus infection. On the day of the transfer, the patient presented again at an external hospital. There had been anuria, vomiting, and severe left-sided leg pain for 2 days. His mean arterial blood pressure was 50 mmHg. Sepsis resulted in acute renal failure with increased retention parameters, a liver synthesis disorder, and deranged coagulation with thrombocytopenia.

The laboratory results revealed increased inflammation: CRP was 274 mg/L, the leucocyte count was 15.5/nL, the ESR was 72 mm/h, and procalcitonin was 78 ng/mL. After 1 day of incubation in anaerobic blood culture, FNE was detected.

**Figure 3:**
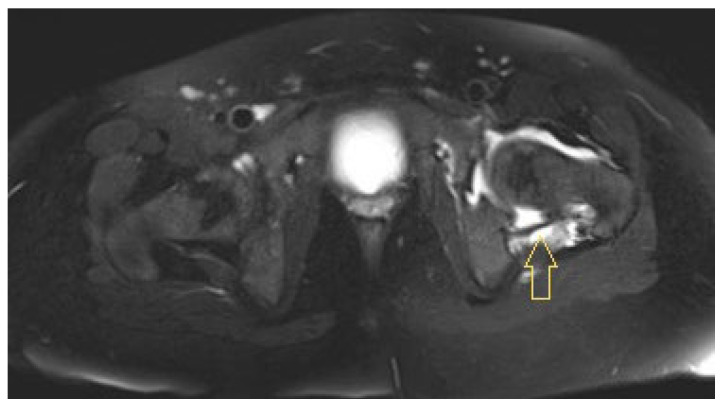
Initial magnetic resonance image of Patient 3: arthritis in the left hip joint and a reaction of the surrounding muscles. Arrow shows to the fluid path.

MRI of the left hip had already been performed at the transferring hospital, which revealed arthritis in the left hip joint with a clear surrounding reaction and fluid in the neighbouring muscles (Figure 3). In addition, a chest X-ray revealed bronchopneumonic infiltrates.

An open arthrotomy of the left hip joint with irrigation and drainage was performed immediately. Anaerobic incubation of tissue from the left hip yielded massive amounts of FNE after 3 days.

Based on the clinical findings, a further joint debridement was indicated 4 days after the initial procedure. The tissue samples no longer showed any evidence of microbes during incubation. Six days later, broad-range 16S rRNA gene PCR results from this tissue were positive for FNE (a 331 bp sequence showed a 100% match to GenBank sequence CP033837).

After 13, 16, and 20 days, additional surgical irrigations were performed due to persistently increased inflammatory parameters, repeated fever episodes, and wound secretion.

**Figure 4:**
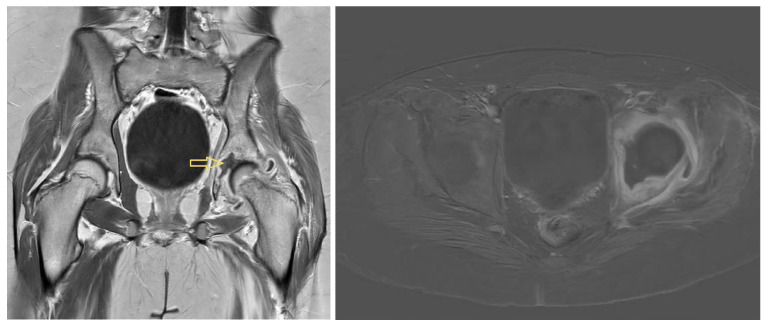
Magnetic resonance images of Patient 3 on hospitalisation day 40: osteomyelitis in the left acetabulum with an abscess in the region of the former Y-joint. Arrow shows to the fluid path.

Positron emission tomography/computed tomography (PET/CT) imaging 1 week after the last joint lavage, performed because of undulating CRP values of approximately 30 mg/L and subfebrile temperatures of approximately 38 °C, revealed the following findings: focal tracer enhancement of both tonsils with a maximum standardised uptake value (SUVmax) of 11.3; cervical primarily reactive lymph nodes (e.g., at the left mandibular angle [SUVmax 11.1]); supleural focal findings on both sides of the lungs (e.g., in the middle lobe [SUVmax 8.0], in the sense of focal infiltrates); and strong focal tracer enhancement of the left hip joint involving the joint capsule (SUVmax 11.7) but also the acetabulum (SUVmax 9.5), with cortical degeneration/decline towards the pelvis. Colleagues from the Ear, Nose and Throat (ENT) Department and the pulmonologists saw no need for further action. Tonsillectomy was not considered to be urgent. MRI at this time revealed clear osteomyelitis in the left acetabulum, with an abscess in the region of the former Y-joint, signs of necrosis of the femoral epiphysis, and joint effusion with synovial thickening consistent with persistent synovitis (Figure 4).

It was again decided to flush the joint via an anterolateral approach and to puncture and flush the bony abscess via a dorsal approach with a strong Jamshidi needle. This occurred 7 weeks after admission to our hospital. PCR and incubation of tissue from this surgery were performed without the detection of microbes.

While the initial symptoms of pain and restricted movement in the left hip joint improved and the wound remained free of irritation, the CRP level stagnated at approximately 50 mg/L, with a normal leucocyte count. The patient walked down the ward corridor, and his fever disappeared.

Antibiotic therapy with piperacillin/tazobactam and clindamycin, which was initiated outside the hospital, was switched to meropenem (1 g IV every 8 h) and vancomycin (600 mg IV every 12 h) in our intensive care unit, and clindamycin (600 mg IV every 8 h) was maintained. Owing to respiratory deterioration on the second day after admission, spotty-infiltrated X-rays and the detection of isolated *Candida* in the tracheal secretions, empirical antimycotic therapy with amphotericin B (100 mg lozenge every 8 h) was given for 4 days due to a suspected fungal infection. One week after admission to our clinic, antibiotic therapy was switched to sultamicillin (Unacid^®^) (3 g IV every 6 h) according to the antibiogram. One week later, this was changed to vancomycin (800 mg IV every 6 h)/meropenem (1 g IV every 8 h) because the patient reacted with severe urticaria when given Unacid^®^. When the central venous catheter had to be removed, this therapy was switched to oral clindamycin (600 mg every 8 h). During the course, this treatment was changed to IV therapy (600 mg every 8 h) for compliance reasons. Because his CRP level was still elevated at 51 mg/L, the antibiotics were changed again to meropenem and vancomycin at the previous dosages and metronidazole (500 mg IV every 8 h). This resulted in the normalisation of the markers of inflammation, and the patient was discharged on HD 73 under this antibiotic therapy, which could be administered at home via a midline catheter. From days 81 to 102 after admission, these antibiotics were changed to oral metronidazole (500 mg every 8 h).

At the last follow-up, 3.7 months after admission to our hospital, the patient felt well. Only his hip rotation was restricted; extension, flexion and abduction were regular. However, an X-ray revealed a significant narrowing of the joint space, consistent with secondary arthrosis, and dense bone structure of the left femoral head, most likely a sign of femoral head necrosis.

### 3.2. Results of the Systematic Literature Review

#### 3.2.1. Report Selection

**Figure 5:**
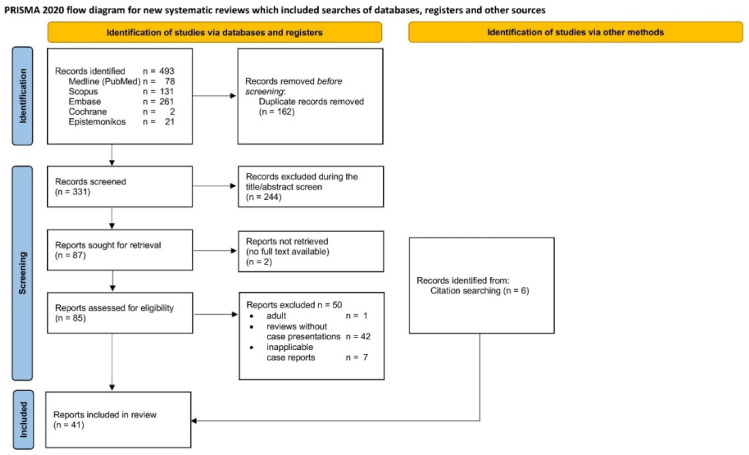
Preferred Reporting Items for Systematic Reviews and Meta-Analyses (PRISMA) flow diagram for systematic literature selection.

The PRISMA flow diagram for article selection is shown in Figure 5. Overall, 41 articles with case presentations of childhood fusobacterial infections of bones and joints were found [10,17,18,19,20,21,22,23,24,25,26,27,28,29,30,31,32,33,34,35,36,37,38,39,40,41,42,43,44,45,46,47,48,49,50,51,52,53,54,55,56]. The reports with cases are listed in Supplementary Table S3. Only three of these reports (Supplementary Table S4) included two or three cases that met the inclusion criteria [53,54,55]. Thus, 45 cases were found in the literature. The reported cases were not included in the data synthesis when they were mentioned only in articles with a different evaluation focus and detailed parameters were not provided (Supplementary Table S5) [1,57,58,59,60,61,62,63,64,65,66,67].

#### 3.2.2. Characteristics of the Reports

Nineteen (42%) of the reports were from the USA; four (9%) were from Canada; three (7%) each were from The Netherlands, Spain, and the UK; and one (2%) each were from Argentina, Australia, Belgium, Finland, France, Germany, India, New Zealand, and Taiwan. The articles were published between 1972 and 2022.

#### 3.2.3. Patient and Microorganism Characteristics

FNU infections were reported in 17 patients (38%), FNE infections were reported in 21 patients (47%), and infections with both *Fusobacterium nucleatum* and *Fusobacterium naviforme* were reported in 1 patient (2%). In six patients (13%), the *Fusobacterium* species were not further specified.

**Figure 6:**
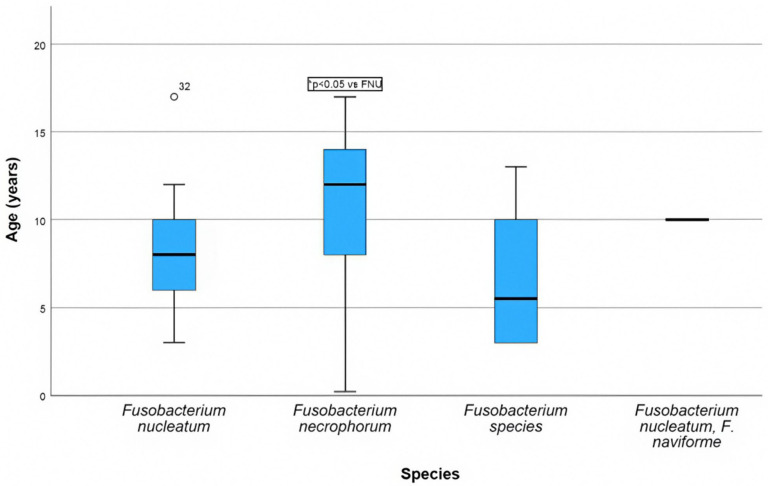
Box plot showing the age of the children depending on the *Fusobacterium* species.

The age of the patients was 9.2 ± 4.1 years, with a median of 9.0 (range 0.2–17.0) years and a mode of 10 years. Children with FNE infections appear to be older than those with FNU infections (10.7 ± 4.4 vs. 8.2 ± 3.3 years, *p* = 0.03, Figure 6). Overall, 33 boys (73%) and 12 girls (27%) were affected; thereby, the risk for boys was 2.75 times higher.

#### 3.2.4. Clinical Presentation

**Figure 7:**
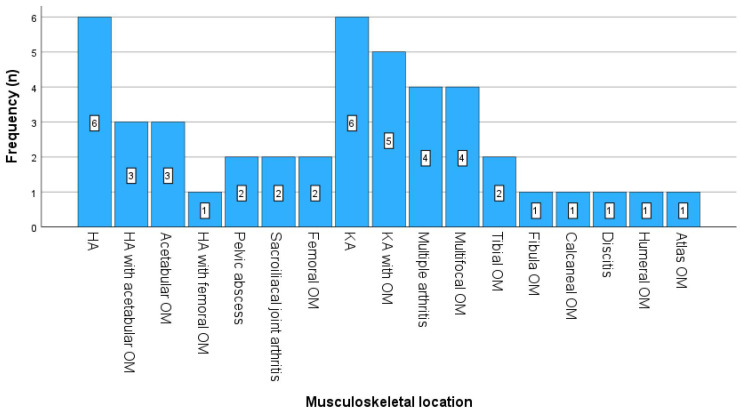
Frequencies of infected joints and bones. Abbreviations: HA, hip arthritis; KA, knee arthritis; OM, osteomyelitis.

Most patients (42%) were affected by hip joint arthritis with or without accompanying acetabular or femoral osteomyelitis and inflammation in the pelvic region. Knee joint arthritis with or without osteomyelitis occurred in 24% of the patients. Multifocal osteomyelitis and arthritis each occurred in 9% of the patients. Furthermore, there were two patients (4%) with tibial osteomyelitis and one patient (2%) each with osteomyelitis of the atlas, humerus, fibula, and calcaneus. Finally, one case (2%) of fusobacterial discitis in childhood has been published (Figure 7).

In 49% of the cases, a focus responsible for musculoskeletal inflammation was found in the ENT region; in 27% of the cases, no focus was found; and in 11%, it was not stated in the publication. Systemic diseases associated with tissue hypoxia (sickle cell disease, Gaucher disease [splenectomised], pulmonary arteriovenous malformation, and respiratory infection) were considered predisposing and were reported in one case each. In one case, the cause was a severely reduced general condition as a result of child abuse.

Patients with an FNE infection were significantly sicker and more severely impaired at initial presentation than patients with an FNU infection (Table 4).

#### 3.2.5. Diagnostics

In 19 cases, the latency between sampling and detection of the microorganism was 5.8 ± 1.5 (range 3–9) days. In patients with an FNU infection, blood cultures were negative, whereas in those with an FNE infection, they were positive in 77% of the patients (*p* = 0.0031). PCR was performed on five patients; it was positive for FNU in all patients. In two cases, the microbe was detected only via PCR (Table 5).

Fourteen arthrocenteses (31%), five abscess aspirations (11%), and four radiologically guided bone biopsies (9%) were performed as diagnostic interventions for the orthopaedic lesions. In fifteen patients (33%), surgical intervention was chosen directly, and in four patients (9%), no information was given on this aspect. In three patients (7%), no invasive diagnostic intervention was performed.

#### 3.2.6. Orthopaedic Surgical Therapy

**Figure 8:**
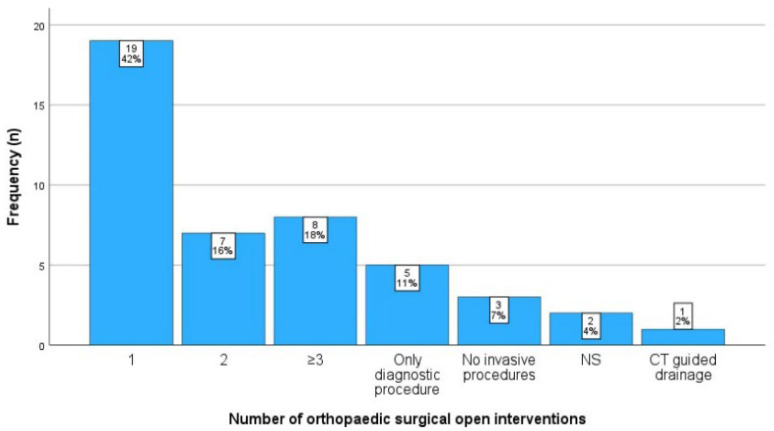
The number of orthopaedic surgical procedures per patient. Abbreviations: CT, computed tomography; ns, not specified.

In 15 patients (33%), arthrotomy was performed, and in 5 patients (11%), arthroscopy was performed. Eleven patients (24%) received bony debridement, and four patients (9%) received soft tissue debridement. Nine patients (20%) did not receive any therapeutic surgical intervention; in one patient (2%), no comment was provided on this issue. The frequency of interventions per patient is shown in Figure 8. In 34% of the patients, multiple surgeries were necessary. The number of recurrent procedures was not significantly different between patients who initially underwent arthrotomy and those who underwent arthroscopy (*p* = 0.303). Furthermore, it did not significantly differ between patients with an FNU infection and patients with an FNE infection (*p* = 0.851).

#### 3.2.7. Antibiotics

Among the 45 patients included, only 1 patient (2%) received oral antibiotic therapy alone. In four patients (9%), antibiotic therapy was not further specified. Of the remaining 40 patients, 21 (52%) received intravenous antibiotic monotherapy after a fusobacterial infection was diagnosed. Eleven patients (28%) were treated with two antibiotics, and eight patients (20%) were treated with three antibiotics. Penicillins, metronidazole, and clindamycin were the most commonly used antibiotics for IV therapy. Twenty-eight patients (62%) received oral antibiotic therapy after IV therapy. No statement was made in this regard for five patients (11%). Clindamycin, metronidazole, and amoxicillin were used most frequently for oral therapy. The antibiotics used for therapy and their frequency of use are summarised in Table 6.

The duration was 3.6 ± 2.0 weeks (range 1–9, reported for 32 patients) for IV antibiotic therapy, 4.7 ± 2.4 weeks (range 1–11, reported for 27 patients) for oral IV therapy, and 7.9 ± 3.1 weeks (range 4–20, reported for 37 patients) for the total duration of antibiotic therapy. Moreover, the duration of antibiotic therapy was 6.7 ± 2.2 weeks for patients with an FNU infection and 8.9 ± 3.8 weeks for patients with an FNE infection (*p* = 0.078).

#### 3.2.8. Outcome

The number of days spent in the hospital was 39.7 ± 34.2 days (range 5–122, reported for 16 patients). The follow-up period was 14.8 ± 12.6 months (range 1.4–48.0, reported for 24 patients). In 38 patients (84%), short- to medium-term outcomes were reported. In 32 patients (71%), healing without sequelae was reported. In one patient each (2%), restricted ROM, severe bone deformity, destruction of the femoral head, coxa magna, neurological deficits, and death were reported. In seven cases (16%), no information on this aspect was provided.

## 4. Discussion

Our case series includes three children aged 11, 12, and 16 years, who were treated at our university hospital over a period of 2 years with osteoarticular manifestations of a fusobacterial infection. The systematic literature review revealed an additional 45 published cases. There are no articles with more than three cases on the topic of osteoarticular fusobacterial infections in children. We synthesised the objective parameters listed in all previously published case reports and thus can make descriptive statements for the first time about a larger patient population.

The following statements regarding osteoarticular fusobacterial infections in children can be made on the basis of the results obtained:

### 4.1. Clinical Course

The clinical characteristics of our three cases correspond to those of the data synthesis from the literature. Almost two-thirds of the cases are boys of approximately 10 years old, whereas the patients with the more virulent FNE [9] are somewhat older. In the majority of patients, the hip joint and the acetabular region are affected. While patients with an FNU infection have minor systemic signs of infection, those with an FNE infection show fulminant courses. It is much easier to detect FNE than FNU. In severely ill septic patients with Lemierre’s syndrome, the microorganisms can usually be detected via anaerobic blood culture. Osteoarticular manifestations are often preceded by an infection in the ENT area. Depending on the affected structure, arthrotomy, arthroscopy, and bone and soft tissue, debridement is used to address the local infection; in more than one-third of cases, the procedures have to be performed several times. Penicillins, metronidazole, and clindamycin are the most commonly used antibiotics and sometimes have to be used for longer than 8 weeks. Despite long hospitalisation times, the outcome in approximately two-thirds of the patients with osteoarticular manifestations was without sequelae, and the clinical findings were good. Nevertheless, in some cases, additional orthopaedic consequences were also present, including restricted ROM, severe bone deformity, and destruction of the femoral head and coxa magna. The latter aspect, often with a good outcome for the joint, possibly distinguishes fusobacterial arthritis from the usual classic development of infectious arthritis caused by *Staphylococcus aureus*, which rapidly leads to irreversible joint damage [24]. Nevertheless, it may be possible to reduce the duration of illness and treatment through reliable and rapid detection of fusobacteria and targeted antibiotic therapy.

### 4.2. Species Detection

Based on our own experience and the systematic literature review, fusobacterial infections in the musculoskeletal system have to be considered rare. However, given that we only detected *Fusobacterium* by PCR in two of our cases and that this is also the case in two other cases in the literature, it must be assumed that *Fusobacterium* species are the cause of osteoarticular infections more frequently than previously assumed. PCR is therefore increasingly recommended for negative cultures in cases of osteoarticular infections. The advantage of PCR is that both dead and viable bacteria may be detected [36,45]. Nevertheless, the correct sampling technique for the cultivation of anaerobic bacteria should be used. Considering the oxygen sensitivity of anaerobic microbes, surgeons should inoculate body fluid directly into anaerobic blood culture media in the operating theatre to enhance laboratory detection [17]. It is essential that all orthopaedic surgeons are knowledgeable about the proper technique to ensure the highest standards for the successful detection of microorganisms. Novel musculoskeletal diagnostic panels specifically designed to provide rapid results for common pathogens in children represent a modern diagnostic option but do not necessarily include the detection of fusobacteria unless it is designed accordingly [68].

### 4.3. Diagnostic Imaging

Another interesting aspect is the radiological appearance on MRI. In Patient 2 of our case series, a bone tumour was initially discussed as a differential diagnosis, something that Budd et al. [21] also described. From our point of view, the acetabular Y-joint seems to be a predilection site from which the infection progresses either into the hip joint or into the pelvis and the surrounding soft tissues. Kokkonen et al. [10] presented similar MR images. In constellations with abscesses around the Y-joint in older children, a Fusobacterium infection should always be considered, even if this cannot be confirmed by imaging alone. On the other hand, in their case series with three FNU infections in knee joint regions, Gregory et al. [54] postulated a possible affinity for the distal femoral epiphysis. Considering the results of our own cases and the systematic review, this should be regarded as a chance finding, as alternatively postulated by Gregory et al. [54]. Last but not least, gas formation has also been described radiologically [29,40]. MRI plays an important role from an orthopaedic point of view for monitoring the local healing process. If abscesses or joint effusions persist after surgical intervention, revision must be performed, despite the fact that clinical parameters may already have normalised. Since fusobacterial infections can also be associated with multiple septic foci, as in Case 3 of our case series, we believe that PET/CT examination is indicated in situations where there is no clinical improvement despite surgical intervention of the known foci and adequate antibiotic therapy.

Antibiotics and additional therapy options. The Red Book of the American Academy of Pediatrics states the following about antibiotic therapy for Fusobacteria infections: ‘*Fusobacterium* species generally are susceptible to metronidazole, clindamycin, chloramphenicol, penicillin with beta-lactamase inhibitor combinations (ampicillin-sulbactam or piperacillin-tazobactam), carbapenems, cefoxitin, and ceftriaxone. Combination therapy with metronidazole or clindamycin in addition to a beta-lactam agent active against aerobic oral and respiratory tract pathogens (e.g., cefotaxime, ceftriaxone, or cefuroxime) is recommended for patients with invasive infection caused by *Fusobacterium* species’ [69].

This means that *Fusobacterium* species may not be adequately covered by first-generation cephalosporins. This is particularly important for the period of empirical antibiotic therapy until species detection. For example, the Children’s Health Queensland Hospital and Health Service guidelines for the treatment of paediatric bone and joint infections recommend flucloxacillin for children over 5 years and cefazolin for children under 5 years [70]. This is similarly described by Donaldson et al., a publication in Pediatrics in Review of the American Academy of Pediatrics [71]. These empirical therapy recommendations are justifiably aimed in particular at *Staphylococcus aureus*, *Streptococcus pyogenes*, and *Kingella kingae* as common pathogens. At this point, it once again becomes clear how important species identification is for the timely implementation of the proposed combination therapy for *Fusobacteria*.

In Patient 3 of our case series, we did not observe a decrease in CRP or fever attacks over a period of approximately 3 weeks. This resulted in repeated surgical interventions on the affected hip. However, at the end of this period, the hip became increasingly clinically unremarkable, and the PCR results of the locally obtained tissue samples were ultimately negative. After we switched the antibiotics from clindamycin, CRP, ESR and fever attacks improved rapidly. This underlines the findings reported in the literature that up to 9% of *Fusobacterium* isolates are resistant to clindamycin and 9% are resistant to penicillin [54,72]. Appelbaum et al. [73] reported β-lactamase production by both FNE and FNU in >20% of isolates.

Another report described additional options with hyperbaric oxygen therapy (HBO) [43]. In everyday clinical practice, antibiotic changes should be considered if there are no signs of clinical improvement. If available, HBO is an additional treatment option in severe cases.

### 4.4. Limitations

Our results must be interpreted carefully due to some limitations. We did not perform a risk of bias assessment of the case reports according to the relevant tools [11,74,75] before inclusion in this review, mainly because we only synthesised objective variables that were not subject to bias. We have not excluded any case reports in order to provide a broad overview of the existing literature in this field. In the results, we always stated the number of missing values. With the objective data provided in the vast majority of reports, a good comparison of the clinical parameters in FNU and FNE could be made (Table 4 and Table 5), but further comparisons, e.g., with regard to antibiotic response and risk factors, were not reliable with the extractable data. The actual bias may arise from cases that have not been published; most likely from cases with poor outcomes. Ultimately, only prospective studies can probably provide sufficient certainty to adequately describe the characteristics of osteoarticular Fusobacterium infections in children. These should be designed in such a way that a sufficiently large patient cohort with positive Fusobacteria detection is generated, e.g., by means of PCR. Only on this basis will it be possible to create or modify treatment protocols and guidelines.

## 5. Conclusions

It can be assumed that paediatric osteoarticular infections with anaerobic *Fusobacterium* species occur more frequently than previously thought. This case series with a systematic review provides guidance for the paediatrician, orthopaedic surgeon, infectious disease specialist, microbiologist, and radiologist who will have first contact with the affected patients on how cases of osteoarticular fusobacterial infections in children usually present and how they should be treated. This should increase awareness of these rather rare infections, and thus, the possibility of a cure without late effects. A typical patient is a boy around the age of 10 years with an infection in the hip area starting from the Y-joint and a previous infection in the ENT area. FNU and FNE are the two most common *Fusobacterium* species responsible for paediatric osteoarticular infections. The latter seems more likely to be virulent and cause severe sepsis, also known as Lemierre’s syndrome. Anaerobic blood culture is usually the most important diagnostic tool for FNE infection. Otherwise, in negative blood cultures and less fulminant conditions, as is the case with FNU infections, the correct collection of anaerobic tissue samples in the operating theatre and the performance of PCR will generally increase the detection of causative microorganisms. The further development and the possibility of using inexpensive and rapid PCR-based diagnostic panels for childhood musculoskeletal infections remain a promising approach. These are prerequisites for effective, targeted antibiotic therapy, and for the healing of the infection without sequelae. In addition, the possibility of resistance to the commonly effective β-lactam antibiotics and clindamycin must be considered.


**Highlights**

Consider osteoarticular fusobacterial infection, especially in older children with pre-vious ENT infection, if no species can be detected with blood and tissue cultures.A correct sampling technique for the cultivation of anaerobic bacteria should be used.Consider PCR/16S ribosomal bacterial DNA amplification to detect the causative microorganisms.Consider an osteoarticular fusobacterial infection if the Y-joint with adjacent hip joint and/or acetabulum is affected.Consider PET/CT to detect further septic lesions if there is no clinical improvement with adequate antibiotic therapy and after surgery.*Fusobacterium nucleatum* and *Fusobacterium necrophorum* are the two most common *Fusobacteria* species responsible for paediatric osteoarticular infections, whereby the clinical course of *F. necrophorum* infection is more severe.Surgical revision is indicated, if in MRI controls, abscesses or joint effusions persist after surgical intervention.Consider during antibiotic therapy, whether a high percentage of *Fusobacteria* produce beta-lactamases and clindamycin resistances are known.


## Figures and Tables

**Table 1:** The PICO (population, intervention, comparison, outcomes) criteria.

Criterion	Description
Population	Children ≤ 18 years with orthopaedically relevant fusobacterial infections
Intervention	Diagnostic measures, as well as orthopaedic surgical and antibiotic treatment
Comparison	None
Outcome	Presentation of the described cases, duration of the disease, and the consequences of the disease on the musculoskeletal system

**Table 2:** Search terms.

Health Condition of Interest		Population
I (#1)	II (#2)	(#3)
Fusobacteri *	arthritis	child *
Fusibacteri *	osteomyelitis	infant *
	osteitis	paediatric
	joint infection	paediatric
	bone infection	
	osseous infection	
	abscess	
	empyema	

**Table 3:** Inclusion and exclusion criteria for title/abstract and full-text screening.

Inclusion Criteria	Exclusion Criteria
Children ≤ 18 years Fusobacterial infections with musculoskeletal involvement	Adults Bone and joint infections with fusobacteria in the throat, nose, ears, and skull region

**Table 4:** Clinical and laboratory findings at initial presentation.

Findings at the Initial Presentation	Patients with a *Fusobacterium necrophorum* Infection	Patients with a *Fusobacterium nucleatum* Infection	*p*-Value
Duration of symptoms until first presentation (days)	9.3 ± 7.8 (n = 18)	20.4 ± 12.0 (n = 14)	0.005
Sepsis	53% (n = 19)	0% (n = 17)	0.014
Fever	100% (n = 19)	47% (n = 15)	0.010
C-reactive protein (mg/L)	202 ± 110 (n = 6)	88 ± 63 (n = 11)	0.007
Erythrocyte sedimentation rate (mm/h)	86 ± 40 (n = 8)	51 ± 25 (n = 13)	0.025
Leucocyte count (10^9^/L)	15.4 ± 8.0 (n = 16)	9.7 ± 3.0 (n = 13)	0.025
Additional lesions *	61% (n = 18)	0% (n = 15)	0.0002

* LMS n = 6, pancytopenia n = 1, epidural abscess n = 1, thrombosis in the internal jugular vein n = 1, endocarditis n = 1, liver abscess n = 1. Number (n) of all patients for whom this statement was provided in the reports.

**Table 5:** Methods for microorganism identification and their results.

Diagnostic Method	Patients with a *Fusobacterium necrophorum* Infection	Patients with a *Fusobacterium nucleatum* Infection	*p*-Value
Blood culture positive/negative (n *)	10/3	0/6	0.0031
Local tissue/fluid positive/negative (n *)	13/1	14/2	0.582
Polymerase chain reaction	Performed in 5 of 45 cases; positive for *F. nucleatum* in all cases		

* Only the patients for whom a statement was provided regarding these parameters were included in the evaluation.

**Table 6:** Frequencies of antibiotics used.

Antibiotic Group	Agent	Used for Intravenous Therapy (n)	Used for Oral Therapy (n)
Penicillins	Penicillin G	12	3
	Amoxicillin	-	7
	Methicillin	1	-
	Nafcillin	1	-
Nitroimidazoles	Metronidazole	13	8
Lincosamides	Clindamycin	10	14
Cephalosporins:			
First-generation	Cefalotin and cefazolin	1 each	-
Second generation	Cefuroxime and cefoxitin	2 each	-
Third generation	Ceftriaxone, cefotaxime, and ceftazidime	1 each	-
	Cefdinir	-	1
Carbapenems	Meropenem Imipenem	6 2	- -
Glycopeptides	Vancomycin	5	-
β-Lactam/β-lactamase inhibitor combinations	Amoxicillin-clavulanate Ticarcillin-clavulanate Ampicillin-sulbactam	1 2 1	- - -
Quinolones	Moxifloxacin Ciprofloxacin	1 1	1 -
Aminoglycosides	Gentamycin	1	-
Amphenicols	Chloramphenicol	1	-
Sulfonamide	Sulfamethoxazole/trimethoprim	-	1

## Data Availability

The original contributions presented in this study are included in the article and Supplementary Materials. Further inquiries can be directed to the corresponding authors.

## Supplementary Materials

The following supporting information can be downloaded at https://www.mdpi.com/article/10.3390/idr17020030/s1. Supplementary Table S1: The detailed search strategy for each database. Supplementary Table S2: Summary of the most important findings of our three patients. Supplementary Table S3: Summary of the case reports that described one case. Supplementary Table S4: Included case series. Supplementary Table S5: Articles mentioning fusobacteria as a causative agent in paediatric bone and joint infections.

## Trial Registration

Centre for Translational and Clinical Research Aachen (CTC-A-No. 24-216), Ethical Committee of RWTH Aachen University (EK 24-420); final statement without reservations from the Risk and Information Governance, University Hospital Aachen, dated 1 October 2024.

## Abbreviations

The following abbreviations are used in this manuscript:

BMI	body mass index
CRP	C-reactive protein
ENT	ear, nose and throat
ESR	erythrocyte sedimentation rate
EUCLIDS	European Union Childhood Life-threatening Infectious Diseases Study
F	female
FNE	*Fusobacterium necrophorum*
FNU	*Fusobacterium nucleatum*
FS	*Fusobacterium* species
FU	follow-up
HA	hip arthritis
HBO	hyperbaric oxygen
HD	hospitalisation day
IV	intravenous
KA	knee arthritis
LMS	Lemierre’s syndrome
M	male
NS	not specified
OM	osteomyelitis
PCR	polymerase chain reaction
PET/CT	positron emission tomography/computed tomography
ROM	range of motion
SUVmax	maximum standardised uptake value

## References

[1] Trobisch A., Schweintzger N.A., Kohlfürst D.S., Sagmeister M.G., Sperl M., Grisold A.J., Feierl G., Herberg J.A., Carrol E.D., Paulus S.C. (2022). Osteoarticular infections in pediatric hospitals in Europe: A prospective cohort study from the EUCLIDS consortium. Front. Pediatr..

[2] Iliadis A.D., Ramachandran M. (2017). Paediatric bone and joint infection. EFORT Open Rev..

[3] Hirschhorn A., Averbuch D., Michaan N., Adler A., Grisaru-Soen G. (2022). Invasive fusobacterium infections in children: A retrospective multicenter study. Pediatr. Infect. Dis. J..

[4] Lemierre A. (1936). On certain septicaemias due to anaerobic organisms. Lancet.

[5] Ullah R., Naz S. (2024). LEMIERRE syndrome: A forgotten infection. J. Ayub. Med. Coll. Abbottabad..

[6] Radovanovic N., Dumic I., Veselinovic M., Burger S., Milovanovic T., Nordstrom C.W., Niendorf E., Ramanan P. (2020). Fusobacterium necrophorum subsp. necrophorum liver abscess with pylephlebitis: An abdominal variant of lemierre’s syndrome. Case Rep Infect Dis..

[7] Jaber F., Alsakarneh S., Alsharaeh T., Salahat A.-J., Elfert K., Beran A., Gangwani M.K., Abboud Y., Al-Sayyed L., Madi M.Y. (2024). Gastrointestinal variant of lemierre’s syndrome: A systematic review and comprehensive analysis of 36 case reports. J. Clin. Exp. Hepatol..

[8] Ibrahim S., Herring J.A. (2022). Tachdjian’s Pediatric Orthopaedics: From the Texas Scottish Rite Hospital for Children.

[9] Bailhache M., Mariani-Kurkdjian P., Lehours P., Sarlangue J., Pillet P., Bingen E., Faye A. (2013). Fusobacterium invasive infections in children: A retrospective study in two French tertiary care centres. Eur. J. Clin. Microbiol. Infect. Dis..

[10] Kokkonen M., Syvänen J., Raitio A., Ivaska L., Peltola V., Helenius I. (2021). Fusobacterial pelvic osteomyelitis with brodie’s abscess in a 10-year-old boy requiring surgical evacuation: A case report. J. Bone Jt. Surg..

[11] Gagnier J.J., Kienle G., Altman D.G., Moher D., Sox H., Riley D. (2013). The CARE guidelines: Consensus-based clinical case reporting guideline development. BMJ Case Rep..

[12] Page M.J., McKenzie J.E., Bossuyt P.M., Boutron I., Hoffmann T.C., Mulrow C.D., Shamseer L., Tetzlaff J.M., Akl E.A., Brennan S.E. (2021). The PRISMA 2020 statement: An updated guideline for reporting systematic reviews. BMJ.

[13] Ouzzani M., Hammady H., Fedorowicz Z., Elmagarmid A. (2016). Rayyan—A web and mobile app for systematic reviews. Syst. Rev..

[14] Singer M., Deutschman C.S., Seymour C.W., Shankar-Hari M., Annane D., Bauer M., Bellomo R., Bernard G.R., Chiche J.-D., Coopersmith C.M. (2016). The third international consensus definitions for sepsis and septic shock (sepsis-3). JAMA.

[15] Murad M.H., Sultan S., Haffar S., Bazerbachi F. (2018). Methodological quality and synthesis of case series and case reports. BMJ Evid. Based Med..

[16] Hiergeist A., Reischl U., Gessner A. (2016). Multicenter quality assessment of 16S ribosomal DNA-sequencing for microbiome analyses reveals high inter-center variability. Int. J. Med. Microbiol..

[17] Almuzam S., Howard-Jones A.R., Birke O., Doyle H., Kesson A.M., Marais B.J. (2021). Subacute osteomyelitis caused by *Fusobacterium nucleatum* in a healthy child. J. Paediatr. Child Health.

[18] Beauchamp R.D., Cimolai N. (1991). Osteomyelitis of the pelvis due to *Fusobacterium nucleatum*. Can. J. Surg..

[19] Beldman T.F.J., Teunisse H.A., Schouten T.J. (1997). Septic arthritis of the hip by Fusobacterium necrophorum after tonsillectomy: A form of Lemierre syndrome?. Eur. J. Pediatr..

[20] Brook I. (2001). Two cases of diskitis attributable to anaerobic bacteria in children. Pediatrics.

[21] Budd E., Johnson D.S., Thomas E., Sadarangani M. (2016). Subacute osteomyelitis of the femur due to *Fusobacterium Nucleatum* in a 7-year-old boy. Pediatr. Infect. Dis. J..

[22] Cubero C.C., Red P.Z., Maestrojuan J.J.S., Prieto M.D.L. (2012). Artritis séptica por *Fusobacterium nucleatum* en paciente inmunocompetente. Reum. Clin..

[23] Chen K.W.K., Chen M.R., Liu S.C., Chiu N.C. (2020). *Fusobacterium* osteomyelitis of the femur in a patient with chronic hypoxemia due to pulmonary arteriovenous malformation: A case report. Pediatr. Neonatol..

[24] Chryssagi A.-M., Brusselmans C.B., Rombouts J.J. (2001). Septic arthritis of the hip due to Fusobacterium *nucleatum*. Clin. Rheumatol..

[25] Clark J.R., Trautwein L.M., Campbell J.R. (2003). An unusual cause of refusal to walk. Semin. Pediatr. Infect. Dis..

[26] Dehority W., Leake J.A. (2008). Fever, Hip Pain, and Headache in a 12-Year-Old Girl. Clin. Pediatr..

[27] Dufillot D., Germaneau J., Taieb A., Fontan D., Guillard J.M. (1986). Septicémie post-angineuse à Fusobacterium necrophorum chez un enfant de 7 ans. [Post-angina septicemia caused by *Fusobacterium* necrophorum in a 7-year-old child]. Arch. Fr. Pediatr..

[28] Epstein M., Pearson A.D., Hudson S.J., Bray R., Taylor M., Beesley J. (1992). Necrobacillosis with pancytopenia. Arch. Dis. Child..

[29] Goyal M., Sharma R., Jain Y., Gupta A., Berry M. (1995). Unusual radiological manifestations of Lemierre’s syndrome: A case report. Pediatr. Radiol..

[30] Gröschel D. (1972). Akute anaerobier-osteomyelitis nach extraktion von milchzähnen. [Acute anaerobes-osteomyelities following extraction of deciduous teeth]. ZWR.

[31] Harris T., Ludwig K.L. (2019). *Fusobacterium necrophorum* pelvic abscess in an adolescent male. J. Investig. Med..

[32] Held M.R., Kotler H., Sneller H., Sullivan C.B. (2018). Lemierre’s Syndrome Presenting as Multifocal Pyomyositis in a Young Child. Pediatr. Infect. Dis. J..

[33] Henry S., DeMaria A., McCabe W.R. (1983). Bacteremia due to Fusobacterium species. Am. J. Med..

[34] Klinge L., Vester U., Schaper J., Hoyer P.F. (2002). Severe Fusobacteria infections (Lemierre syndrome) in two boys. Eur. J. Pediatr..

[35] Koornstra J.J., Veenendaal D., A Bruyn G., de Graaf H. (1998). Septic arthritis due to *Fusobacterium nucleatum*. Rheumatology.

[36] Kroon E., Arents N.A., Halbertsma F.J. (2012). Septic arthritis and osteomyelitis in a 10-year-old boy, caused by *Fusobacterium nucleatum*, diagnosed with PCR/16S ribosomal bacterial DNA amplification. BMJ Case Rep..

[37] de la Hoz J.A., del Río P.G., García M.d.M.B., Artuch N.M., Martín M.J.R. (2021). Osteomielitis por anaerobios. Una Entid. Poco Frec. Pediatr..

[38] Luey C., Tooley D., Briggs S. (2012). Emphysematous osteomyelitis: A case report and review of the literature. Int. J. Infect. Dis..

[39] Litterio M., Soto A., Aguirre C., Uranga M., Rubeglio E. (2004). Lemierre’s syndrome: Case report in a pediatric patient. Anaerobe.

[40] Liu A.C., Argent J.D. (2002). Necrobacillosis—A Resurgence?. Clin. Radiol..

[41] Lovse L.J., Coupal S.A., Tice A.D.W., Le Saux N., Carsen S.P. (2021). Pediatric Acetabular Osteomyelitis Treated with Hip Arthroscopy. JAAOS Glob. Res. Rev..

[42] Masterson T., El-Hakim H., Magnus K., Robinson J. (2005). A case of the otogenic variant of Lemierre’s syndrome with atypical sequelae and a review of pediatric literature. Int. J. Pediatr. Otorhinolaryngol..

[43] Murray S.J., Lieberman J.M. (2002). Fusobacterium osteomyelitis in a child with sickle cell disease. Pediatr. Infect. Dis. J..

[44] Naylor A.R., Briggs M.S., Kegelmeyer D.K., Kloos A.D. (2013). Rehabilitation and functional outcomes after extensive surgical debridement of a knee infected by fusobacterium necrophorum: A case report. Int. J. Sports Phys. Ther..

[45] Rojo-Martin M.D., Miranda C., Torres E., Sabalete T., Mazuelas P. (2009). Septic arthritis caused by anaerobic bacteria. Clin. Microbiol. Infect..

[46] Schubiner H., Letourneau M., Murray D. (1981). Pyogenic Osteomyelitis Versus Pseudo-Osteomyelitis in Gaucher’s Disease. Clin. Pediatr..

[47] Seidenfeld S.M., Sutker W.L., Luby J.P. (1982). Fusobacterium necrophorum septicemia following oropharyngeal infection. JAMA.

[48] Sonsale P.D., Philipson M.R., Bowskill J. (2004). Septic Arthritis of the Knee Due to *Fusobacterium necrophorum*. J. Clin. Microbiol..

[49] Stahlman G.C., DeBoer D.K., Green N.E. (1996). Fusobacterium Osteomyelitis and Pyarthrosis: A Classic Case of Lemierre’s Syndrome. J. Pediatr. Orthop..

[50] Trapp C.M., Tamai J., Schleiss M.R. (2005). Septic arthritis secondary to fusobacterium necrophorum in a 4-year-old girl: Case report and review of the literature. Pediatr. Infect. Dis. J..

[51] Trutner Z., Hwang R., Bowen R. (2022). Pediatric Septic Arthritis of the Knee due to Fusobacterium necrophorum in a Patient with Down Syndrome. J. Bone Jt. Surg..

[52] Van Dyke D.C., Alexander R.C., Perlman S., Smith W.J., Dekowski S.A. (1989). Fusiform Bacterial Sepsis. Clin. Pediatr..

[53] Vogel L.C., Boyer K.M. (1980). Metastatic Complications of Fusobacterium necrophorum Sepsis. Am. J. Dis. Child..

[54] Gregory S.W., Boyce T.G., Larson A.N., Patel R., Jackson M.A. (2015). *Fusobacterium nucleatum* Osteomyelitis in 3 Previously Healthy Children: A Case Series and Review of the Literature. J. Pediatr. Infect. Dis. Soc..

[55] Rathore M.H., Barton L.L., Dunkle L.M. (1990). The spectrum of fusobacterial infections in children. Pediatr. Infect. Dis. J..

[56] Sabella C., Kuivila T., Andrish J., Hall G., Goldfarb J. (2001). Fusobacterial Pyogenic Arthritis in Two School-Aged Children. Infect. Dis. Clin. Pr..

[57] Abbati G., Abu Rumeileh S., Perrone A., Galli L., Resti M., Trapani S. (2022). Pelvic Pyomyositis in Childhood: Clinical and Radiological Findings in a Tertiary Pediatric Center. Children.

[58] Reigosa B.A., López I.M.C., Marcos E.S., Galán-Olleros M., Olivier M.S., Gero L.C., García R.J. (2024). Hospital at Home Program for the Treatment of Pediatric Osteoarticular Infections. Hosp. Pediatr..

[59] Ballock R.T., Newton P.O., Evans S.J., Estabrook M., Farnsworth C.L., Bradley J.S. (2009). A Comparison of Early Versus Late Conversion from Intravenous to Oral Therapy in the Treatment of Septic Arthritis. J. Pediatr. Orthop..

[60] Brook I. (1986). Msc Anaerobic osteomyelitis in children. Pediatr. Infect. Dis. J..

[61] Brook I. (1998). Aerobic and anaerobic microbiology of infections after trauma in children. Emerg. Med. J..

[62] Filleron A., Laurens M.E., Marin G., Marchandin H., Prodhomme O., Alkar F., Godreuil S., Nagot N., Cottalorda J., L’kaissi M. (2019). Short-course antibiotic treatment of bone and joint infections in children: A retrospective study at Montpellier University Hospital from 2009 to 2013. J. Antimicrob. Chemother..

[63] Jagodzinski N.A., Kanwar R., Graham K., Bache C.E. (2009). Prospective Evaluation of a Shortened Regimen of Treatment for Acute Osteomyelitis and Septic Arthritis in Children. J. Pediatr. Orthop..

[64] Juchler C., Spyropoulou V., Wagner N., Merlini L., Dhouib A., Manzano S., Tabard-Fougère A., Samara E., Ceroni D. (2018). The Contemporary Bacteriologic Epidemiology of Osteoarticular Infections in Children in Switzerland. J. Pediatr..

[65] Li C.N., Nakamura M.M. (2022). Utility of Broad-Range PCR Sequencing for Infectious Diseases Clinical Decision Making: A Pediatric Center Experience. J. Clin. Microbiol..

[66] Ogden J.A., Light T.R. (1979). Pediatric osteomyelitis: III. anaerobic microorganisms. Clin. Orthop. Relat. Res..

[67] Serrano E., Ferri I., Galli L., Chiappini E. (2020). Amoxicillin-clavulanic acid empirical oral therapy for the management of children with acute haematogenous osteomyelitis. Antibiotics.

[68] Searns J.B., Robinson C.C., Wei Q., Yuan J., Hamilton S., Pretty K., Donaldson N., Parker S.K., Dominguez S.R. (2019). Validation of a novel molecular diagnostic panel for pediatric musculoskeletal infections: Integration of the cepheid xpert MRSA/SA SSTI and laboratory-developed real-time PCR assays for clindamycin resistance genes and kingella kingae detection. J. Microbiol. Methods.

[69] Kimberlin D.W., Banerjee R., Barnett E.D., Lynfield R., Sawyer M.H. (2024). Red Book: 2024–2027 Report of the Committee on Infectious Diseases: Fusobacterium Infections (Including Lemierre Syndrome).

[70] Children’s Health Queensland Hospital and Health Service. Paediatric Bone and Joint Infection Management: Guideline. https://www.childrens.health.qld.gov.au/__data/assets/pdf_file/0029/176915/guide-paed-bone-jnt.pdf.

[71] Donaldson N., Sanders J., Child J., Parker S. (2020). Acute Hematogenous bacterial osteoarticular infections in children. Pediatr. Rev..

[72] Aldridge K.E., Ashcraft D., Cambre K., Pierson C.L., Jenkins S.G., Rosenblatt J.E. (2001). Multicenter survey of the changing in vitro antimicrobial susceptibilities of clinical isolates of *Bacteroides fragilis* group, *Prevotella*, *Fusobacterium*, *Porphyromonas*, and *Peptostreptococcus* species. Antimicrob. Agents Chemother..

[73] Appelbaum P.C., Spangler S.K., Jacobs M.R. (1990). Beta-lactamase production and susceptibilities to amoxicillin, amoxicillin-clavulanate, ticarcillin, ticarcillin-clavulanate, cefoxitin, imipenem, and metronidazole of 320 non-Bacteroides fragilis Bacteroides isolates and 129 fusobacteria from 28 U.S. centers. Antimicrob. Agents Chemother..

[74] Munn Z., Barker T.H., Moola S., Tufanaru C., Stern C., McArthur A., Stephenson M., Aromataris E. (2020). Methodological quality of case series studies: An introduction to the JBI critical appraisal tool. JBI Evid. Synth..

[75] Moola S., Munn Z., Tufanaru C., Aromataris E., Sears K., Sfetcu R., Currie M., Qureshi R., Mattis P., Lisy K., Aromataris E., Munn Z. (2020). Systematic reviews of etiology and risk. JBI Manual for Evidence Synthesis.

