# Supplementary material for: Fusobacterium Species in Osteoarticular Infections in Childhood—A Systematic Review with Data Synthesis and a Case Series in the Acetabular and Hip Joint Regions
Source: Infect Dis Rep. 2025 Apr 10;17(2):30. doi: 10.3390/idr17020030 (PMC12026919; doi:10.3390/idr17020030)
Supplement: Supplementary file 1 [file idr-17-00030-s001.zip › Supplemantary Table S3 Summary of the case reports that described one case.pdf]

Supplementary Table 3 Summary of the case reports that described one case

	Age (years)	Gender	Musculoskeletal location	Presumed original focus	Sepsis [12]	Fever* (≥38°C)	Further lesions	C-reactive protein (mg/l)*	Erythrocyte sedimentation rate (mm/h)*	Leucocyte count (10^9/l)*	Duration of symptoms** (days)	Species	Day of identification after taking samples	Blood culture	Local tissue/fluid	Polymerase chain reaction	Orthopaedic interventions/number	Day of intervention***	Main antibiotics/duration (weeks)****	Length of hospitalisation (days)	Final outcome regarding orthopaedic lesion	Follow-up (months)
Almuzam et al., 2021 Sydney, Australia [17]	12	M	HA with acetabular OM	Not known	No	No	No	85	104	8.2	28	FNU	6	-	+	NS	Arthrotomy/1	3	Moxifloxacin/6	NS	CA	2
Beauchamp et al., 1991 Vancouver, BC, Canada [18]	6	M	Acetabular OM	Not known	No	Yes	No	NS	48	6.9	30.4	FNU	NS	NS	+	NS	Bony site aspirated under fluoroscopic guidance	NS	Clindamycin IV/4 clindamycin oral/6	NS	CA	3
Beldman et al., 1997 Nieuwegein, Netherlands [19]	9	M	HA	Tonsils	No	Yes	No	360	50	25.4	1	FNE	4	+	+	NS	Arthrotomy/2	1<br>7	Penicillin G iv/2<br>Clindamycin oral/3	14	CA	NS
Brook, 2001	8	M	Diskitis L3/L4	Pharyngitis	No	Yes	No	ns	42	11,0[+]	10	FNU	NS	NS	+	NS	CT-guided	NS	Clindamycin IV/3	NS	Complete resolution	24

Washington, USA [20]																	aspiration		clindamycin oral/3			
Budd et al., 2015 Vancouver, BC, Canada [21]	7	M	KA with OM	Not known	No	Yes	No	200	55	9.7	42	FNU	NS	-	+	+	Arthrotomy	NS	Clindamycin, penicillin G IV/2 Clindamycin oral/4	NS	Full recovery	24
Carrasco Cubero et al., 2012 Cádiz, Spain [22]	6	M	KA with OM	Otitis	No	Yes	No	124	30	5.5^{+}	14	FNU	NS	-	+	NS	Arthrotomy	NS	Metronidazole IV/3 metronidazole oral/2	NS	CA	NS
Chen et al., 2020 Taipei, Taiwan [23]	14	F	Femur OM	Chronic hypoxemia due to pulmonary arteriovenous malformation	No	NS	NS	262	NS	16.9	21	FNE	NS	NS	+	NS	Sequestrectomy, debridement	NS	Vancomycin/3 clindamycin/2	NS	No recurrence of OM	48
Chryssagi et al., 2001 Louvain and Brussels, Belgium [24]	9	M	HA	Not known	No	No	No	78	68	11.4	18	FNU	9	NS	+	NS	Arthrotomy, drain for 5 days	2	Cefuroxime IV/2 amoxycillin oral/3	NS	Slight coxa magna	33
Clark et al., 2003 Houston, TX, USA [25]	3	M	Calcaneal OM	Not known	No	No	No	NS	23	11.9	7	FS	NS	-	+	NS	Surgical exploration	8	Clindamycin, cefoxitin IV/8	10	CA	6

Dehority et al., 2008 San Diego, CA, USA [26]	12	F	Gluteal abscesses of both sides	Otitis	Yes	Yes	LMS	158	NS	14.5	7	FNE	NS	NS	+	NS	Drainage of abscesses	2	Meropenem IV/6 amoxicillin oral/4	21	CA	5
Dufillot et al., 1986 Bordeaux, France [27]	7	M	HA with femoral OM	Tonsils	Yes	Yes	LMS	NS	125	28.6	30.4	FNE	NS	+	-	NS	Irrigation-aspiration drainage of the femur	NS	Penicillin G IV/NS	NS	Destruction femoral head	NS
Epstein et al., 1992 Newcastle upon Tyne, UK [28]	5	F	Osteitis humerus	Otitis	Yes	Yes	Pancytopenia	NS	NS	19.0	7	FNE	6	+	NS	NS	Radiological diagnosis/ no surgical therapy	-	Penicillin, metronidazole, imipenem/8	42	Severe deformity of the arm	NS
Goyal et al., 1995 New Delhi, India [29]	8	M	HA with acetabular OM	Pharyngitis	Yes	Yes	LMS, gas surrounding the pelvic organs	NS	NS	14.0	2	FNE	NS	+	+	NS	Arthrocentesis	NS	NS	NS	Succumbed to septicaemia	NS
Gröschel, 1972 Springfield, MA, USA [30]	11	F	Femoral OM	Tooth extraction	Yes	Yes	No	NS	110	18.2	21	FNE	NS	NS	+	NS	Bone abscess drainage, further several orthopaedic surgeries	1	Methicillin and cefalotin IV/NS	122	NS	NS

Harris et al., 2019 Edmont, OK, USA [31]	15	M	Pelvic abscess	Pharyngitis	No	Yes	No	NS	NS	NS	12	FNE	NS	-	+	NS	Drain under CT guidance	NS	Ceftriaxone, vancomycin and metronidazole IV/2 Cefdinir, metronidazole, sulfamethoxazole/trimethoprim oral/4	14	NS	NS
Held et al., 2017 Hartford, CT, USA [32]	4	M	KA, HA, diffuse inflammatory myopathy throughout both lower extremities, clavicle abscess	Otitis	Yes	Yes	LMS	242	115	7.6	16	FS	6	-	+	NS	Debridement of abscesses in left lateral thigh, left posterior hamstring, left calf and right thigh	1 3 NS	Vancomycin, ceftazidime and metronidazole/NS Metronidazole and clindamycin oral/4	29	NS	NS

Henry et al., 1983 Boston, MA, USA [33]	17	M	Multifocal OM	Pharyngitis	No	NS	NS	NS	NS	NS	NS	FNU	NS	NS	NS	NS	NS	NS	NS	NS	NS	NS
Klinge et al., 2002 Essen, Germany [34]	13	M	Atlas and clivus OM with gaseous inclusions	Otitis	Yes	NS	LMS	300	NS	20.0	NS	FS	8	+	NS	NS	No		Meropenem, vancomycin and gentamycin/4 Penicillin oral/4	70	CA	2.3
Kokkonen et al., 2021 Turku, Finland [10]	10	M	Acetabular OM, abscess over quadrilateral plate	Not known	No	Yes°	No	166	NS	11.3	2	FNU and FNA	NS	-	+	+	Ilioinguinal approach, surgical evacuation	3	Cefuroxime, clindamycin and metronidazole IV/1 Amoxicillin–clavulanic acid oral/2 Clindamycin oral/4	8	CA	12
Koornstra et al., 1998 Leeuwarden, Netherlands [35]	10	M	Ka	Not known	No	No	No	155	NS	5.5^{+}	21	FNU	6	NS	+	NS	Arthrocentesis	NS	Penicillin and metronidazole IV/4	28	NS	NS
Kroon et al., 2012 Veldhoven, Netherlands [36]	10	M	Ha	Not known	No	No	No	46	69	7.4	NS	FNU	NS	-	-	+	Arthrocentesis	NS	Amoxicillin/clavulanate IV/2 Clindamycin oral/6	NS	Discharged in good condition	NS

La Alonso de la Hoz et al., 2021 Madrid, Spain [37]	10	M	HA with acetabular OM	Not known	No	No	No	152	NS	13.6	35	FNU	7	NS	-	+	Initial arthroscopy on day 5 and arthrotomy on day 7	1	Clindamycin, metronidazole IV/3 Amoxicillin–clavulanate oral/3	NS	CA	24
Luey et al., 2012 Auckland, New Zealand [38]	15	F	Emphysematous OM sacrum and os ilium with soft tissue abscess	Tonsillitis and road accident	Yes	Yes	Epidural abscess	Yes	Yes	3.9	13	FNE	NS	+	+	NS	Debridement of abscesses on days 1, 3, 5 and 7	1	Clindamycin, metronidazole IV/2 Penicillin IV/2 Penicillin oral/4	105	Bilateral leg weakness, self-catheterisation and manual bowel evacuation	18
Litterio et al., 2004 Buenos Aires, Argentina [39]	0.2	M	OM tibia and humerus	Otitis	Yes	Yes	LMS	NS	NS	8.3	7	FNE	6	+	+	NS	Surgical drainage	NS	Meropenem iv/2 amoxicillin-clavulanate oral/6	36	CA	6
Liu et al., 2002 Southampton, UK [40]	15	F	KA with lower leg abscess, pyomyositis of vastus intermedius	Pharyngitis	No	Yes	Thrombosis in the internal jugular vein, gas in the calf	225	NS	11.9	NS	FNE	5	NS	+	NS	Arthrocentesis, abscess drainage and arthroscopy	NS	Metronidazole and cefotaxime	42	Residual flexion deformity of the knee	1.4

Lovse et al., 2021 London, Ontario, Canada [41]	10	F	Acetabular OM	NS	Nos	No	NS	150	37	11.3	21	FS	6	NS	+	NS	Joint aspiration on day 9 and arthroscopy on day 11	1	Cefazolin, meropenem IV/4 Metronidazole oral/9	NS	CA and full return to sport	24
Masterson et al., 2005 Edmonton, Canada [42]	8	F	Distal fibula OM	Otitis	Yes	Yes	LMS	NS	NS	NS	3	FNE	3	NS	+	NS	Soft tissue abscess drainage	NS	Metronidazole IV/3 metronidazole oral/3	NS	NS	NS
Murray et al., 2002 Long Beach, CA, USA [43]	7	M	Proximal tibia OM	Sickle cell disease	No	No	No	27	30	15.6	30.4	FNU	NS	NS	+	NS	Debridement infected bone	21	Clindamycin IV/6 hyperbaric oxygen treatment	NS	Resumed full activities	1.5
Naylor et al., 2013 Columbus, OH, USA [44]	17	F	KA, HA, abscesses thigh and calf	Pharyngitis volleyball trauma	No	Yes	No	NS	NS	NS	8	FNE	6	NS	+	NS	Arthrocentesis, arthrotomy and debridement gluteal thigh and calf musculature (8 surgeries)	NS	Vancomycin, metronidazole and ciprofloxacin IV Clindamycin, metronidazole and amoxicillin oral	NS	Return to competitive softball	8

Rojo-Martin et al., 2009 Granada, Spain [45]	3	M	KA	Not known	No	NS	NS	NS	NS	NS	NS	FNU	NS	NS	+	+	Arthrotomy	NS	Imipenem, metronidazole iv/ns clindamycin oral/ns	NS	Satisfactory	NS
Schubiner et al., 1981 East Lansing, MI, USA [46]	7	F	Proximal tibia OM	Gaucher's disease (splenectomised)	No	Yes	No	NS	55	16.2	4	FS	NS	-	+	NS	Surgical drainage	1	Nafcillin IV/2 clindamycin oral/4		CA	3
Seidenfeld et al., 1982 Dallas, TX, USA [47]	13	F	KA, ankle arthritis, foot OM	Pharyngitis	No	Yes	Endocarditis and septic emboli based on chest X-ray	NS	NS	32.4	3	FNE	7	+	+	NS	Arthrotomy	4	Penicillin G IV/3 cefoxitin iv/6	NS	Complete recovery	NS
Sonsale et al., 2004 Derby, UK [48]	8	M	KA	Dental abscess	No	Yes	No	NS	23	10.5	2	FNE	6	NS	+	NS	Arthrotomy and drainage	1	Metronidazole oral/ns	NS	CA	30
Stahlman et al., 1996 Nashville, TN, USA [49]	13	M	OM left ankle and right knee	Pharyngitis	No	Yes	Pneumonia and LMS	NS	65	15.4	10	FNE	NS	NS	+	NS	Abscess aspiration, abscess debridement	NS	Penicillin iv/6	NS	Complete recovery	NS

Trapp et al., 2005 Cincinnati and Minneapolis, MN, USA [50]	4	F	KA	Not known	No	Yes	No	34	65	5.6	NS	FNE	NS	-	+	NS	Arthrotomy/3	1	Ampicillin–sulbactam IV/3 Due to exacerbation of infection: meropenem and clindamycin IV/6	NS	Good functional outcome	1.5
Trutner et al., 2022 Los Angeles, CA, USA [51]	16	M	KA with OM	Down syndrome and blunt trauma	No	Yes	No	173	122	12.0	9	FNE	NS	-	+	NS	Arthrocentesis, arthroscopy with limited synovectomy	NS	Clindamycin iv/1 clindamycin oral/11	5	CA	12
Van Dyke et al., 1989 Iowa City, IA, USA [52]	3	M	OM distal femur both sides, left prox. tibia	Munchausen by proxy, child abuse suspected	Yes	Yes	Hepatic abscesses	NS	35	11.5	5	FS	NS	+	NS	NS	Multiple surgical procedures	NS	NS	NS	NS	NS
Vogel et al., 1980 Chicago, IL, USA [53]	12	M	Arthritis left shoulder, hip, knee	Tonsilitis	Yes	Yes	Liver enlargement and encephalopathy	ns	125	9.2	5	FNE	NS	+	NS	NS	No		Penicillin G, Chloramphenicol IV/3 Penicillin V oral/9	28	Symptoms resolved completely	NS

*at initial presentation; ** prior initial presentation; *** after initial presentation; ****after the microorganism became known; + age-appropriate normal value [59]. Abbreviations: CA, clinically asymptomatic; CT, computed tomography; F, female; FNA, *Fusobacterium naviforme*; FNE, *Fusobacterium necrophorum*; FNU, *Fusobacterium nucleatum*; FS, *Fusobacterium* species; HA, hip arthritis; IV, intravenous; KA, knee arthritis; LMS, Lemierre's syndrome; M, male; NS, not specified; OM, osteomyelitis; ROM range of motion
