# Supplementary material for: Fusobacterium Species in Osteoarticular Infections in Childhood—A Systematic Review with Data Synthesis and a Case Series in the Acetabular and Hip Joint Regions
Source: Infect Dis Rep. 2025 Apr 10;17(2):30. doi: 10.3390/idr17020030 (PMC12026919; doi:10.3390/idr17020030)
Supplement: Supplementary file 1 [file idr-17-00030-s001.zip › Supplementary Table S1 The detailed search strategy.pdf]

Supplementary Table 1 The detailed search strategy for each database

**Database**	**Combinations of search words with Boolean operators**
MEDLINE (PubMed)	("Fusobacterium nucleatum"[MeSH] OR "Fusobacterium"[All Fields]) AND ("Children"[MeSH] OR "Child"[All Fields]) AND ("Bone Infections"[MeSH] OR "Osteomyelitis"[MeSH] OR "Joint Diseases"[MeSH] OR "Empyema"[MeSH] OR "Osteitis"[All Fields])
Embase	('Fusobacterium nucleatum'/exp OR 'Fusobacterium'/exp) AND ('children'/exp OR 'pediatric'/exp OR 'infant'/exp) AND ('bone infection'/exp OR 'osseous infection'/exp OR 'osteomyelitis'/exp OR 'joint infection'/exp OR 'arthritis'/exp OR 'empyema'/exp OR 'osteitis'/exp OR 'abscess'/exp)
Scopus	(TITLE-ABS-KEY("Fusobacterium nucleatum") OR TITLE-ABS-KEY("Fusobacterium")) AND (TITLE-ABS-KEY("Children") OR TITLE-ABS-KEY("Child")) AND (TITLE-ABS-KEY("Bone infection") OR TITLE-ABS-KEY("Osteomyelitis") OR TITLE-ABS-KEY("Joint infection") OR TITLE-ABS-KEY("Empyema") OR TITLE-ABS-KEY("Osteitis"))
Epistemonikos	(("Fusobacterium nucleatum" OR "Fusobacterium" OR "F. nucleatum") AND ("Children" OR "Child" OR "Pediatric" OR "Infant") AND ("Bone infection" OR "Osteomyelitis" OR "Joint infection" OR "Empyema" OR "Osteitis" OR "Abscess" OR "Arthritis"))
CENTRAL	("Fusobacterium nucleatum" OR "Fusobacterium" OR "F. nucleatum") AND ("Children" OR "Child" OR "Pediatric" OR "Infant") AND ("Bone infection" OR "Osteomyelitis" OR "Joint infection" OR "Empyema" OR "Osteitis" OR "Abscess" OR "Arthritis")
