# Supplementary material for: Fusobacterium Species in Osteoarticular Infections in Childhood—A Systematic Review with Data Synthesis and a Case Series in the Acetabular and Hip Joint Regions
Source: Infect Dis Rep. 2025 Apr 10;17(2):30. doi: 10.3390/idr17020030 (PMC12026919; doi:10.3390/idr17020030)
Supplement: Supplementary file 1 [file idr-17-00030-s001.zip › Supplementary Table S2 Summary of our three patients.pdf]

Supplementary Table 2 Summary of the most important findings of our three patients

	**Patient 1**	**Patient 2**	**Patient 3**
Age (years)	11	12	16
Gender	Female	Male	Male
Region	Right hip joint and acetabulum	Right acetabulum, abscess in the pelvis	Left hip joint and acetabulum
Height; weight	1.44 m; 36.4 kg	1.62 m; 63 kg	1.75 m; 84.5 kg
Presumed original focus of infection	Severe diarrhoea	Not known	Tonsils
**Initial clinical findings at admission**			
Fever	37.5°C	37.5°C	41°C
Septic	No	No	Yes
**Laboratory findings at first presentation in hospital**			
C-reactive protein (mg/l)	45	40	274
Leucocytes/nl	9.3	8.8	15.5
Erythrocyte sedimentation rate (mm/h)	72	114	72
Procalcitonin (ng/ml)	Not specified	Not specified	78

**Magnetic resonance imaging findings**			
	Arthritis of the right hip joint with bony involvement and abscess formation extending into the gluteal soft tissues	Extensive osteodestructive process in the right os ilium/ischiadicum with cortical destruction at the level of the medial acetabular wall and posterior acetabular pillar<br>Melting at the level of the epiphyseal groove and subperiosteally on the medial acetabular wall, measuring up to 1.5 and 4.2 cm	Initial:<br>arthritis in the left hip joint with a clear surrounding reaction and fluid in the neighbouring muscles<br>Hospitalisation day 40:<br>clear osteomyelitis in the left acetabulum with an abscess in the region of former Y-joint, signs of necrosis of the femoral epiphysis and still joint effusion with synovial thickening consistent with persistent synovitis
**Surgery**			
Type of initial surgery	Hospitalisation day 1: arthrotomy with irrigation and drainage	Hospitalisation day 2: fenestration of the abscess in the Y-joint using a 7 mm hollow reamer via the first window ilioinguinal approach, introduction of gentamycin loaded calcium sulfate beads into the abscess cavity and the reaming channel	Hospitalisation day 1: arthrotomy with irrigation and drainage<br>A total of 6 irrigations on the left hip joint
Special procedures during the course of treatment	Hospitalisation day 4: extensive capsular fenestration with irrigation and the removal of the abscess on the dorsal acetabulum with the insertion of a gentamycin sponge	None	Hospitalisation day 41: flushed the joint from an anterolateral approach and punctured and flushed the bony abscess from a dorsal approach using a strong Jamshidi needle
Hospitalisation day of surgery	1 and 4	2	1, 5, 18, 20, 24 and 41
**Microorganism characteristics**			
Microorganism	*Fusobacterium nucleatum*	Most likely *F. nucleatum* (see polymerase chain reaction row)	*Fusobacterium necrophorum*
Blood culture	Hospitalisation day 1: only aerobes – negative<br>Hospitalisation day 7: aerobes and anaerobes – negative	Not carried out	Positive after 1 day of incubation

Culture of local tissue/fluid	Negative	Negative	*F. necrophorum* en masse
Polymerase chain reaction	Positive	100 % match with the GenBank sequences for *F. nucleatum*, *Fusobacterium animalis* and *Fusobacterium vincentii*	*F. necrophorum*; a 333 base pair sequences showed a 100% match with GenBank sequence pf058913
Time until microorganism became known	21 days	11 days	1 day
**Antibiotic therapy**			
Calculated	Cefuroxime intravenous for 5 days<br>Ceftriaxone and clarithromycin intravenous for 16 days	Clindamycin and ampicillin–sulbactam intravenous for 8 days<br>Ampicillin–sulbactam intravenous for 8 days	Piperacillin/tazobactam + clindamycin intravenous
Targeted (mainly)	Clindamycin intravenous for 9 days<br>Clindamycin oral for 2 weeks	Clindamycin oral/4 weeks	Hospitalisation days 2–8: meropenem, vancomycin and clindamycin intravenous<br>Hospitalisation days 8–16: ampicillin–sulbactam intravenous<br>Hospitalisation days 16–37: meropenem and vancomycin<br>Hospitalisation days 37–60: clindamycin intravenous<br>Hospitalisation days 60–73: meropenem, vancomycin and metronidazole<br>After discharge:<br>meropenem, vancomycin and metronidazole intravenous for 8 days, followed thereafter by metronidazole oral for 3 weeks
**Outcome/follow-up**			
Days in hospital	29	17	73
Follow-up (months)	17.7	6.1	3.7
Last clinical findings	No restricted range of motion and no complaints	No restricted range of motion and no complaints	Restricted hip rotation; otherwise, no complaints

Last radiological findings	Magnetic resonance imaging: residual post-inflammatory structural irregularities in the dorsocranial right acetabulum; no florid inflammatory changes in the pelvis and hip joints; no femoral head necrosis	Magnetic resonance imaging: almost complete regression of the previously extensive inflammatory-abscessing bone marrow changes in the right acetabulum with still minor, reactive changes; no florid osteomyelitis; no abscesses	X-ray: narrowing of the joint space, consistent with secondary arthrosis; dense bone structure of the left femoral head, most likely indicative of femoral head necrosis.
