# Supplementary material for: Fusobacterium Species in Osteoarticular Infections in Childhood—A Systematic Review with Data Synthesis and a Case Series in the Acetabular and Hip Joint Regions
Source: Infect Dis Rep. 2025 Apr 10;17(2):30. doi: 10.3390/idr17020030 (PMC12026919; doi:10.3390/idr17020030)
Supplement: Supplementary file 1 [file idr-17-00030-s001.zip › Supplementary Table S4 Included case series.pdf]

Supplementary Table 4 Included case series

	Age (years)	Gender	Musculoskeletal location	Presumed original focus	Sepsis [12]	Fever* (≥38°C)	Further lesions	C-reactive protein (mg/l)*	Erythrocyte sedimentation rate (mm/h)*	Leucocyte count (10^9/l)*	Duration of symptoms** (days)	Species	Day of identification after taking samples	Blood culture	Local tissue/fluid	Polymerase chain reaction	Orthopaedic interventions/number	Day of intervention***	Main antibiotics/duration (weeks)****	Length of hospitalisation (days)	Final outcome regarding orthopaedic lesion	Follow-up (months)
Gregory et al., 2015 Rochester, USA [54]	6	M	KA with OM	NS	No	No	No	3	9	10.7	4	FNU	7	NS	+	NS	Arthrotomy/2	1	Metronidazole IV/4 Amoxicillin–clavulanate oral/2	ns	CA	ns
	7	M	KA with OM	NS	No	Yes	No	26	18	10.9	28	FNU	NS	NS	+	NS	Arthroscopy/2	1	Metronidazole/12	ns	CA	ns
	4	M	KA	NS	No	Yes	No	69	64	10.1	10	FNU	3	NS	+	NS	Arthroscopy	ns	Penicillin IV/1 Clindamycin oral/8	ns	CA	ns

Rathore et al., 1990 St. Louis, MO, USA [55]	9	M	HA	pharyngitis	Yes	Yes	NS	NS	NS	NS	6	FNE	NS	+	+	NS	Hip drainage	ns	Penicillin IV/6 Clindamycin oral/4	60.8	No sequelae	18
	14	M	HA	NS	NS	NS	NS	NS	NS	NS	NS	FNE	NS	+	+	NS	Hip drainage and several surgical procedures	ns	4.5 months	ns	Complete recovery	ns
Sabella et al., 2001 Cleveland, OH, USA [56]	10	M	HA	Respiratory infection	No	Yes	No	NS	70	NS	10	FNU	5	-	+	NS	Arthrotomy	1	Ticarcillin–clavulanate/6 No oral antibiotics	ns	Remained well	24
	8	M	Sacroiliac joint arthritis	Not known	No	No	No	NS	52	NS	5	FNU	5	-	+	NS	Aspiration of the sacroiliac joint under fluoroscopic guidance	ns	Ticarcillin–clavulanate/4 No oral antibiotics	ns	Remained well	24

*at initial presentation; ** prior initial presentation; *** after initial presentation; ****after the microorganism became known; [+] age-appropriate normal value [59]. Abbreviations: CA, clinically asymptomatic; FNA, *Fusobacterium naviforme*; FNE, *Fusobacterium necrophorum*; FNU, *Fusobacterium nucleatum*; FS, *Fusobacterium* species; HA, hip arthritis; IV, intravenous; KA, knee arthritis; LMS, Lemierre's syndrome; M, male; NS, not specified; OM, osteomyelitis; ROM range of motion
