# Supplementary material for: Fusobacterium Species in Osteoarticular Infections in Childhood—A Systematic Review with Data Synthesis and a Case Series in the Acetabular and Hip Joint Regions
Source: Infect Dis Rep. 2025 Apr 10;17(2):30. doi: 10.3390/idr17020030 (PMC12026919; doi:10.3390/idr17020030)
Supplement: Supplementary file 1 [file idr-17-00030-s001.zip › Supplementary Table S5 Articles mentioning fusobacteria.pdf]

Supplementary Table 5 Articles mentioning fusobacteria as a causative agent in paediatric bone and joint infections

**Author, year, origin and reference number**	**Topic of the article**	**Reference to Fusobacteria**
Abbati et al., 2022 Florence, Italy [57]	47 children with pelvic pyomyositis	Of 23 cases (48.9%) in which a causative pathogen could be identified, *Fusobacterium necrophorum* was found based on polymerase chain reaction of drained pus in a 16.5-year-old patient with Lemierre's syndrome.
Agúndez et al., 2024 Madrid, Spain [58]	Characteristics of 88 patients with confirmed paediatric osteoarticular infections who required intravenous antibiotic therapy received as part of a hospital-at-home programme	Of 101 samples (26 synovial fluid, 75 blood culture), one synovial fluid sample was positive for *Fusobacterium nucleatum.*
Ballock et al., 2009 San Diego, CA, USA [59]	186 patients from two children's hospitals with septic arthritis	*F. nucleatum* was detected in one case.
Brook et al., 1986 Bethesda, MD, USA [60]	26 paediatric patients with osteomyelitis caused by anaerobic bacteria	An orthopaedically relevant case report on an 11-year-old boy with a mixed infection of the metacarpal bones with FNU, *Peptococcus* sp. and streptococci after a dog bite
Brook et al., 1998 Bethesda, MD USA [61]	175 specimens obtained from 166 children with trauma	Detection of *Fusobacteria* spp. with possible orthopaedic relevance: once for myositis, twice for osteomyelitis and once on the finger
Filleron et al., 2019 Montpellier, France [62]	Evaluation of a short-term antibiotic protocol for both osteomyelitis and septic arthritis in 176 children	The aetiological agent was identified in 42% of the cases; in one case, it was *F. nucleatum.*
Jagodzinski et al., 2009 Birmingham, UK, and Melbourne, Australia [63]	Evaluation of a shortened regimen of treatment for acute osteomyelitis and septic arthritis in 70 children	One case with *Fusobacterium* in aspirate culture in septic arthritis

Juchler et al., 2018 Geneva, Switzerland [64]	Bacteriologic epidemiology of paediatric osteoarticular infections with particular regard to children's ages in 217 children	One case of *Fusobacterium canifelinum* in the group '6–48 months of age'
Naureckas Li et al., 2022 Boston, MA, USA [65]	382 broad-range polymerase chain reaction samples were identified from 269 unique paediatric patients: utility in clinical decision-making	A total of 19 of 382 (5.0%) samples resulted in a change in clinical management; 1 case of *F. necrophorum* in shoulder
Ogden et al., 1979 Haiti [66]	Nine paediatric cases of anaerobic osteomyelitis	One case had distal humerus osteomyelitis with *Bacteroides fragilis* and *Fusobacterium fusiforme*.
Serrano et al., 2019 Florence, Italy [67]	153 children with acute haematogenous osteomyelitis who underwent at least one microbiological test to highlight the extent of the use and the possible role of amoxicillin–clavulanate in the oral treatment	At least one organism was isolated in 37.9% (58/153) cases.<br><br>One case with *F. necrophorum* in pus/biopsy polymerase chain reaction
Trobisch et al., 2022 EUCLIDS Consortium [1]	380 patients with paediatric osteoarticular infections; a causative organism was detected in 65% (247/380)	One case with *F. necrophorum*

Abbreviation: EUCLIDS, European Union Childhood Life-threatening Infectious Diseases Study
